# Supplementary material for: The consequences of using different epoch lengths on the classification of accelerometer based sedentary behaviour and physical activity
Source: PLoS One. 2021 Jul 15;16(7):e0254721. doi: 10.1371/journal.pone.0254721 (PMC8282067; doi:10.1371/journal.pone.0254721)
Supplement: Attachment — Submitted filename: Response-to-reviewers.docx [file pone.0254721.s002.docx]

PONE-D-19-08551
Be aware of the consequences of scaling your cut-point to different epoch lengths

**Journal Requirements:**
When submitting your revision, we need you to address these additional requirements.

1. Please ensure that your manuscript meets PLOS ONE's style requirements, including those for file naming. The PLOS ONE style templates can be found at
   <http://www.journals.plos.org/plosone/s/file?id=wjVg/PLOSOne_formatting_sample_main_body.pdf> and <http://www.journals.plos.org/plosone/s/file?id=ba62/PLOSOne_formatting_sample_title_authors_affiliations.pdf>

AUTHOR’S REPLY

We adjusted the style requirements according to the provided style templates.

2. Please modify the title of your article so that it is specific, descriptive, concise, and comprehensible to readers outside the field (as recommend in the journals submission guidelines).Please ensure that you amend both the title on the online submission form (via Edit Submission) and the title in the manuscript so that they are identical.

AUTHOR’S REPLY

We have revised the title as follows:

*The consequences of using different epoch lengths on the classification of accelerometer-based sedentary behaviour and physical activity.*

Additionally, we have revised the short title as follows:

*Consequences of using different epoch lengths*

3. We note that you have indicated that data from this study are available upon request. PLOS only allows data to be available upon request if there are legal or ethical restrictions on sharing data publicly. For information on unacceptable data access restrictions, please see http://journals.plos.org/plosone/s/data-availability#loc-unacceptable-data-access-restrictions.In your revised cover letter, please address the following prompts:
   a. If there are ethical or legal restrictions on sharing a de-identified data set, please explain them in detail (e.g., data contain potentially identifying or sensitive patient information) and who has imposed them (e.g., an ethics committee). Please also provide contact information for a data access committee, ethics committee, or other institutional body to which data requests may be sent.
   b. If there are no restrictions, please upload the minimal anonymized data set necessary to replicate your study findings as either Supporting Information files or to a stable, public repository and provide us with the relevant URLs, DOIs, or accession numbers. Please see http://www.bmj.com/content/340/bmj.c181.long for guidelines on how to de-identify and prepare clinical data for publication. For a list of acceptable repositories, please see <http://journals.plos.org/plosone/s/data-availability#loc-recommended-repositories>.

AUTHOR’S REPLY

For the SOS-data, there are no restrictions. We have included this data as Supporting Information file. For the CHAMPS-data, restrictions are in place, which we now explained:

”The data supporting the conclusions of this article are stored in the Danish National Archives. Data are available upon request from the CHAMPS-DK steering committee, as the data as determined by the University’s legal department is not allowed to be stored on a public server due to legal and ethical restrictions. Interested parties may contact Dr. Niels Wedderkopp (nwedderkopp@health.sdu.dk). The following information is mandatory at the time of application: a description of how data will be used, securely managed and stored, and finally permanently deleted”.

**Reviewer #1:** The present study examined the impact of two different epoch lengths on total time and bouts spent sedentary and in physical activity. Although the study is generally sound and uses relatively strong datasets, especially for free-living activity, several issues need to be improved.

AUTHOR’S REPLY

We thank the reviewer for thoroughly reviewing our manuscript and providing valuable suggestions to improve our manuscript. Please find below our point-to-point reply.

1) The study compares 15- and 16-second epochs, whereas previous studies have suggested that using even shorter epochs is favourable. As the datasets used for the present analysis have data in 1- and 2-second epoch lengths, why is not a shorter epoch included, for example 2-second? The study concludes with a recommendation to use 15-second epoch, but this conclusion should be drawn after also evaluating shorter epochs. Thus, I strongly recommend also including a 2-second epoch duration to be able to make a better-informed conclusion.

AUTHOR’S REPLY

We thank the reviewer for this suggestion and have now included a shorter epoch duration, i.e. of 5-seconds, to both the analysis of the SOS-data (classification accuracy of sedentary behaviour) and the CHAMPS-data (free-living time estimates of sedentary behaviour and physical activity). We have revised our manuscript based on these additional analyses.

2) The title of the paper should be revised. The title suggests that the problem evaluated is the scaling of the cut points. Obviously, the cut points used to classify intensity-specific PA needs to be scaled when using different epochs. However, using different epoch settings is a well-known challenge. Thus, the use of different epoch settings, not scaling of cut points, is the issue of the paper, and should be reflected in the title.

AUTHOR’S REPLY

We have revised the title as follows:

*The consequences of using different epoch lengths on the classification of accelerometer-based sedentary behaviour and physical activity.*

Additionally, we have revised the short title as follows:

*Consequences of using different epoch lengths*

3) Regarding the SOS-study, it seems strange to evaluate classification accuracy, after excluding the behaviour that is not of interest, for example by excluding non-sedentary time when examining accuracy for sedentary time, which means the dataset only includes the positive cases or intervals, but not the negative. Moreover, I believe it would be easier for readers if results are reported as time spent in different intensities for the different epoch settings, instead of percentages (Table 2).

AUTHOR’S REPLY

The reviewer is correct that the SOS-dataset only included positive cases/intervals. To assess the classification accuracy (of sedentary behaviour; first study aim) we designed a controlled-laboratory study where we imposed the (mainly sedentary) activities to be sure that the imposed activities were actually performed. In a few situations, the participants did not adhere to the protocol and we excluded these time intervals. By including one standing and one dancing activity and calculating the percentage of time during these activities that were classified as sedentary, the dataset also included negative intervals. For more details, see pages 6-7, lines 125-145 (study design) and pages 10-11 lines 228-237 (statistical analyses).

Table 2 refers to the accuracy of classifying sedentary behaviour and MVPA, where 100% indicates all time periods correctly classified. We believe that providing percentages is more precise/accurate when depicting these results, and therefore prefer keeping percentages.

4) The study cites many relevant studies, but the study by Aadland et al. (2018). "Associations of volumes and patterns of physical activity with metabolic health in children: A multivariate pattern analysis approach." Preventive Medicine 115: 12-18 is lacking. Of importance for the present study, this study evaluated the use of different epoch durations (1, 10, and 60 seconds) to determine bouts of intensity-specific physical activity across bout durations from 1 second to more than 60 minutes, which would provide an important perspective for interpretation of the present findings. Line 100 should be revised when including this study.

AUTHOR’S REPLY

We thank the reviewer for mentioning this interesting study, which we have now included the introduction and discussion.

- We referred to this study in our introduction and discussion.
- We summarized the results of this study in the introduction, lines 76-82:

*Nettlefold et al [16] and Aadland et al [19] examined the influence of epoch length on bouts of physical activity and sedentary behaviour of different durations. Both studies found more time accumulated in MVPA bouts of various durations with increasing epoch lengths, i.e. more time in MVPA bouts ranging between 5 and ≥20 minutes [16] and VPA and MPVA bouts ranging between 3 and 29 minutes [19]. Additionally, both studies found more time accumulated in bouts of sedentary behaviour and LPA with increasing epoch lengths, especially in bouts ranging from 5-10 minutes [16] and 5-29 minutes [19].*

- We referred to the findings of this study in the discussion, lines 382-384, lines 399-404 and 409-410:

*- Previous studies found lower, higher and similar estimates of total MPA and MVPA time [11-17, 19, 21, 22] and lower estimates of total VPA time [11, 13-16, 19, 21, 22] with increasing epoch lengths.*

*- Four previous studies examined the influence of epoch length on bouts of MPA and MVPA [11, 16, 17, 19]. Aibar et al [17] and Aadland et al [19] found lower estimates of MVPA in bouts of ≥10 minutes [17] and lower estimates of MPA, VPA and MVPA in bout durations between 1.5-1.9 minutes and ≥60 minutes [19] when applying higher epoch lengths (i.e. 60 versus 3 seconds [17] or 60 versus 1 seconds [19]).*

*- Moreover, differences may be explained by different tolerance times in bouts, as demonstrated by Aadland et al [19].*

Line 100 (in the current manuscript lines 111-113) refers to studies examining the influence of epoch length on the accuracy of classifying accelerometer-derived sedentary behaviour, compared to direct observation. We have revised this sentence to clarify this:

*To date, studies examining the influence of epoch length on the classification accuracy of accelerometer-derived sedentary behaviour are lacking.*

5) Statistics: Please specify how differences in time spent in various intensities between epoch settings were tested – did this test account for the repeated measurements (e.g., paired-samples t-test?). I am surprised that many comparisons were not significant since I would believe the effect of using different epoch settings would be rather similar for all individuals, which means relatively small differences would be significant.

AUTHOR’S REPLY

We critically checked all data and decided to use non-parametric statistics, as most data were not normally distributed. We used medians and 25^th^-75^th^ percentiles to present the data and the Mann-Whitney U test to calculate differences between the different epoch durations, i.e. 5- vs. 15-second epochs, 5- vs. 60-second epochs and 15- vs. 60-second epochs. We carefully checked all numbers and revised our manuscript based on these new analyses:

- Methods, statistical analyses (page 11, lines 237-239): *Using CHAMPS-data, as time estimates according to 5-, 15- and 60-second epoch lengths were not normally distributed, medians (25th - 75th percentiles) were calculated and tested for significance in R using Mann-Whitney U (wilcox.test, R platform 3.4.0; p<0.05).*
- Results: See page 16, lines 289-305.
- Results: Table 3 (pages 17-18) and Figure 1.

6) Discussion line 284-: This paragraph question to what extent the activities applied is suitable as a criterion for evaluation of accelerometry. If dancing is regarded MVPA, but there are several periods standing still, only parts of the session is MVPA. The accelerometer, which captures movement, will correctly capture these periods as not being of moderate or vigorous intensity. Thus, I ask the authors to consider this point and make sure this perspective is reflected in the paper.

AUTHOR’S REPLY

We agree that the periods of time where children and adolescents were standing still to choose and start an new dance, could be accurately classified as not being moderately active. However, we did not mark these short standing periods, nor is our measure of heart rate valid to identify these periods. Therefore, we can only conclude on the time that was misclassified as sedentary. We have revised this paragraph as follows (lines 356-365):

*As we did not mark these short standing periods and these periods may not necessarily have led to a lower heart rate (i.e. based on heart rate: 34% and 49% of dancing time classified as moderate intensity), we could not assess whether the accelerometer accurately classified these periods as not being at a moderate intensity. However, it is surprising that zero percent of dancing time was classified as MVPA when applying a cut point of ≥2296 count per minute, while applying a cut-point of ≥574 counts per 15 seconds and ≥192 counts per 5 seconds resulted in 20% (children) to 29-33% (adolescents) of dancing time classified as MVPA. This may suggests that especially a 60-second epoch fails to detect MVPA during dancing: a 60-second epoch may sum physical activity at various intensities, thereby not detecting short bursts of MVPA.*

7) Discussion line 361-: Please state/speculate why these differential patterns are found for total time versus bouts. As argued by the authors, the use of shorter epoch leads to greater sensitivity to capture sporadic bursts of activity and thus variation in intensity from epoch to epoch, which is the reason why shorter epochs lead to less consecutive time in bouts.
AUTHOR’S REPLY

We have revised the discussion section based on the additional analyses in a shorter epoch duration (i.e. 5 seconds) and the non-parametric statistics, and included potential explanations for findings that were in contrast to previous study findings.

8) Please include error bars on the figures.
AUTHOR’S REPLY

We have added error bares to the figures.

**Reviewer #2:** Dear authors
This is well-written paper presenting the investigation of the contribution of the epoch-lengths (15s vs 60s) on physical activity intensity output (SED, LPA, MPA, VPA) on both lab-setting and free-living in children.

AUTHOR’S REPLY

We thank the reviewer for thoroughly reviewing our manuscript and providing valuable suggestions to improve our manuscript. Please find below our point-to-point reply.

There are some major issues I would like to comment on here:

It seems natural that when applying a longer epoch would reduce the possibility to capture the variation of SED, MPA and VPA in children and they will turn into the LPA group. Using bouts would add another factor that reduce the possibility to capture variation in children in the same variables. A suitable activity that indicates this is the dancing activity that one could assume is a more intermittent activity. From the free-living data one could also observe that with 15s epochs the time was in general higher for the shortest bout and for the total time for SED, MPA, VPA but not LPA. Studies have shown that children would have even shorter periods of activity, i.e. <10s (Aadland 2018). The question is: is it really warranted to recommend using 15s epochs when not discussing these results in relation to studies that have shown that children’s´ activities can be even shorter bursts? If researchers now apply 15s epoch and still miss time in physical activity, then we would continue with measurement errors. I would suggest that you discuss your results in relation to the more recent findings, e.g. Aadland 2018. I can understand that this recommendation is in relation to the older accelerometers such as GT3X, but today newer models and devices are used. A do agree that it is important with methodological investigations to inform other researchers, but I question the contribution if it still does not target the measurement error with capturing the intermittent or spontaneous nature of children.
AUTHOR’S REPLY

We thank the reviewer for mentioning this interesting study, which we have now included the introduction and discussion:

- We summarized the results of this study in the introduction, lines 78-80:

*Nettlefold et al [16] and Aadland et al [19] examined the influence of epoch length on bouts of physical activity and sedentary behaviour of different durations. Both studies found more time accumulated in MVPA bouts of various durations with increasing epoch lengths, i.e. more time in MVPA bouts ranging between 5 and* ≥*20 minutes [16] and VPA and MPVA bouts ranging between 3 and 29 minutes [19]. Additionally, both studies found more time accumulated in bouts of sedentary behaviour and LPA with increasing epoch lengths, especially in bouts ranging from 5-10 minutes [16] and 5-29 minutes [19].*

- We referred to the findings of this study in the discussion, lines 382-384, lines 399-404 and 409-410:

*- Previous studies found lower, higher and similar estimates of total MPA and MVPA time [11-17, 19, 21, 22] and lower estimates of total VPA time [11, 13-16, 19, 21, 22] with increasing epoch lengths.*

*- Four previous studies examined the influence of epoch length on bouts of MPA and MVPA [11, 16, 17, 19]. Aibar et al [17] and Aadland et al [19] found lower estimates of MVPA in bouts of ≥10 minutes [17] and lower estimates of MPA, VPA and MVPA in bout durations between 1.5-1.9 minutes and ≥60 minutes [19] when applying higher epoch lengths (i.e. 60 versus 3 seconds [17] or 60 versus 1 seconds [19]).*

*- Moreover, differences may be explained by different tolerance times in bouts, as demonstrated by Aadland et al [19].*

Moreover, we agree that including a shorter epoch duration further strengthens our study. We have included an epoch duration of 5-seconds to both the analysis of the SOS-data (classification accuracy of sedentary behaviour) and the CHAMPS-data (free-living time estimates of sedentary behaviour and physical activity). We have revised our manuscript based on these additional analyses.

I would also like the authors to provide a rational to the use of and investigate on 10min bouts in children, on MPA and especially on VPA. Is it because just of tradition or is it of real importance to study it in children? It is likely that children do not move continuously in 10 minutes, especially not running. I would like the author to develop the discussion of the meaning of applying the 10min bout in capturing the physical activity behavior of children. Is it just another type of epoch or what do we do when we apply the 10min bout criterium?
AUTHOR’S REPLY

Thus far, there is no consensus on what bout duration of physical activity and sedentary behaviour are relevant for health. Previous studies suggest that MVPA (MPA, VPA) accumulated in bouts may have different effects on children’s health, when compared to the total volume of MVPA (Poitras et al, Appl Physiol Nutr Metab, 2016; Cliff et al, Obes Rev, 2016; Mark & Janssen, Am J Prev Med, 2009; Willis et al, Prev Med, 2015; Dorsey et al, Int J Pediatr Obes, 2011). When examining bouts of MVPA, many studies regard periods of time less than 5 minutes as ‘sporadic’ or unbouted PA . As children rarely spent their MVPA in bouts longer than 10 minutes (Baquet et al, Prev Med, 2007; Chinapaw et al, MSSE, 2019), we extracted bouts of 5-10 as well as bouts longer than 10 minutes. We agree with the reviewer that including bouts of more than 10 minutes might not be useful in children. Yet we included both bout durations to provide a complete overview and to be able to compare the occurrence of MVPA bouts of different durations as well as the influence of epoch duration on these bout durations.

As the current study examines the methodological issue of selecting an epoch length when analysing accelerometer data, and does not include any associations with children’s health, we cannot contribute to the discussion on relevant bout durations of physical activity and sedentary behaviour. Therefore, we prefer to keep the manuscript focus on the effects of epoch duration.

Interestingly, the high misclassification of SED as LPA in the first study with 15s epochs and by camera it was observed that the children is no sitting still. Wouldn´t then 15s epoch be too long as the children are not sedentary? I would think that the part of sedentary that we really want to investigate is when we are sitting still and the effect on cardiometabolic health.
AUTHOR’S REPLY

We would like to point out that the finding of using a 15-second epoch resulted in a larger misclassification when compared to a 60-second epoch indicates that the 15-second epoch is too short to accurately classify sedentary behaviour. In a previous article we demonstrated that children rarely sit still (van Ekris et al, 2018, IJERPH). Therefore, in our opinion, examining the health effects of sitting still is not reflecting children’s behaviour and not meaningful to study.

Minor comments
In the introduction (page 4 line 53) and in the method (page 7, line 145), you call the 1s counts as raw accelerometer data. I suggest that you do not call it raw data as researchers working with real raw acceleration data would not agree. You can call it as it is, second-by-second counts.

AUTHOR’S REPLY

We thank the reviewer for this suggestion and have changed ‘raw data’ into ‘second-by-second data’ throughout the manuscript.

In the method section, page 8, line 150: As an accelerometer register the instant acceleration, while the HR monitor record the following but delayed physiological response, using HR as reference for the activity intensity during dancing (intermittent) is not optimal, unless the children performed the same movement intensity across the whole activity. In this case, both the test method and the reference method have their errors which might be commented on as it may affect the outcome of your study.

AUTHOR’S REPLY

We agree with the reviewer that using heart rate is not an optimal for determining the intensity of physical activities. We have added this to the limitations paragraph, lines 463-465:

*Third, the use of heart rate monitoring to estimate the intensity of the dancing activity is a limitation of our study, as heart rate provides a delayed record of the physiological response.*

**Reviewer #3:** Thanks for the opportunity to review the manuscript entitled “Be aware of the consequences of scaling your cut-point to different epoch lengths” submitted to PLOS ONE. Using data from two studies, the aims of the paper are to examine the influence of scaling accelerometer cut-points on the classification accuracy of laboratory-controlled sedentary behavior, and free-living total time and time spent in bouts of sedentary behavior and moderate-to-vigorous physical activity among youth. The manuscript will be a contribution to researchers and practitioner within the field of sedentary behavior and physical activity. Below are my comments that hopefully will improve the manuscript further.

AUTHOR’S REPLY

We thank the reviewer for thoroughly reviewing our manuscript and providing valuable suggestions to improve our manuscript. Please find below our point-to-point reply.

Broad comments:
Throughout the manuscript, the authors alternates between using the terms “sedentary behavior” and “sedentary time”. It could be argued that the term “sedentary behavior” reflect some types of behaviors (e.g., screen-time) whereas “sedentary time” reflect the total sedentary time accumulated across different domains. Since accelerometers are used in the present study, the term “sedentary time” most accurately reflect what is being measured and estimated, especially in the CHAMPS-study DK. The authors might consider revising accordingly.

AUTHOR’S REPLY

We critically checked the manuscript and revised sedentary behaviour into sedentary time where appropriate.

The authors are recommended to sync the aims provided in the abstract, at the end of the introduction and at the beginning of the discussion. Currently, both “youth”, “children and adolescents”, “children”, and “primary school children” are used. Similarly, both the terms “sedentary behavior” and “sedentary time” are used.

AUTHOR’S REPLY

We thank the reviewer for pointing us at this inconsistency in wording. We carefully checked the manuscript and omitted the wordings ‘primary school children’ and ‘youth’ throughout the text, and replaced this with ‘children’, ‘adolescents’ or ‘children and adolescents’.

Moreover, we revised sedentary behaviour into sedentary time where appropriate.

Some of the reference cited (e.g., on page 4, lines 67-70) involve both children and adolescent (e.g., Edwardson et al.). In its present form, only “children” is used when referring to these references. The authors might consider using both “children” and “adolescents”.

AUTHOR’S REPLY

We revised this – where appropriate – according to the reviewer’s suggestion.

Given that the CHAMPS-study DK involved children aged 5-12 years, and that no differences were observed when comparing the two epoch lengths during free-living sedentary time and physical activity, it might be interesting to at least comment whether or not the results were similar among younger and older children.

AUTHOR’S REPLY

In response to the comments of reviewer #1 and #2, we added a third, shorter epoch duration (i.e. 5 seconds) to the comparison. The reviewer poses an interesting additional research question. Since the manuscript already contains a lot of information and analyses (i.e. data from two different studies, analysed using 3 different epoch durations), we prefer not to include an additional research question, analyses and results.

The discussion is sometimes challenging to follow due to the multiple perspectives and references cited. I recommend the authors to re-arrange the discussion to highlight the main findings of the study.

AUTHOR’S REPLY

Following a paragraph on the main findings, we highlighted first the findings regarding our first aim (based on the controlled-laboratory study) and subsequently the findings regarding our second aim (based on data from the CHAMPS-study). The discussion is completed with a paragraph on the strengths and limitations and the conclusions. We revised the text to improve the readability.

The fact that only one physical activity was included in the SOS study is a limitation. Although mentioned, this limitation should be highlight and discussed more thoroughly.

AUTHOR’S REPLY

We would like to point out that we referred to this limitation throughout the discussion section:

- Pages 19-20, lines 338-339: *It should be noted that we only included one light-to-moderate (dancing) activity.*
- Page 21, lines 370-378: *An explanation for these different findings may be the physical activities included in the different study protocols. The SOS-study included only one physical activity (i.e. dancing) which based on heart rate was classified as moderate intensity 34-49% of time, and which was occasionally interrupted with standing still. Apparently this dancing activity included short periods of moderate intensity activity, which were detected using 5- and 15-second epochs but not using a 60-second epoch. The children in the study of McClain et al [12] participated in a range of physical activities during physical education class. The activities during physical education may have induced a more constant level of moderate-to-vigorous intensity of longer duration detected by the applied 10-60-second epoch lengths.*
- Page 23, lines 437-440: *We recommend future studies evaluating the classification accuracy of cut-points and the potential influence of epoch length to include activities covering the full spectrum of sedentary behaviour and physical activity at various intensities.*

Specific comments:
Page 4, line 60-62. The statement in relation to cut-points by Puyau et al. should be supported by references.
AUTHOR’S REPLY

We revised this sentence to make clear that we included this as an example what we mean with scaling cut-points to match a specific epoch duration (page 3, lines 55-58):

*For example, the Puyau cut-point of ≥3200 counts per minute for classifying moderate-to-vigorous physical activity (MVPA) can be scaled to match a 15-second epoch, resulting in a cut-point of ≥800 counts per 15-seconds.*

Page 5, line 91. As “PE” is used later in the manuscript, it should be abbreviated when first mentioned and then further used at page 7, line 132, and page 18, line 302.
AUTHOR’S REPLY

We thank the reviewer for this comment and have revised this as suggested.

Page 5, line 94-99. It is stated that “by including direct observation as comparison measure, McClain et al. could not only examine differences in accelerometer-derived MVPA, but also conclude on the most accurate epoch length for assessing MVPA”. As the authors refer to McClain and colleagues’ findings that 5 seconds epoch length “resulted in the smallest deviations from observation-based MVPA”, the reader might wonder why the authors choose 15 seconds epoch length as part of their own analyses. Thus, it is recommended to provide arguments for choosing 15 seconds epoch length.

AUTHOR’S REPLY

In response to the comments of the other reviewers, we now included a shorter epoch duration, i.e. of 5-seconds, to both the analysis of the SOS-data (classification accuracy of sedentary behaviour) and the CHAMPS-data (free-living time estimates of sedentary behaviour and physical activity). Therefore, instead of providing arguments for choosing a 15-second epoch duration, we added the 5-second epoch duration. We have revised our manuscript based on these additional analyses.

Page 5, line 99-100. It is stated that “to date, studies examining the influence of epoch length on accelerometer-derived sedentary behavior are lacking”. Is this statement related to direct observations, i.e., there are lack of studies examining the influence of epoch length on accelerometer-derived sedentary behavior by direct observations? Please clarify this because the statement could also be read as studies examining the influence of epoch length on accelerometer-derived sedentary behavior are lacking overall (which appears contradictory given the multiple references cited at page 4, line 69).

AUTHOR’S REPLY

The reviewer is correct that we are referring here to studies examining the influence epoch length on the classification accuracy of sedentary behaviour. We have revised this sentence, lines 111-113:

*To date, studies examining the influence of epoch length on the classification accuracy of accelerometer-derived sedentary behaviour are lacking*.

Page 10, lines 218-219 and page 11, lines 220-235. The percentages mention do not fully correspond to the figures provided in table 2 (or the other way around). For example, when using 15 seconds epoch length: a) In table 2, the sedentary activity “gaming” is not 95.7 % but 96.0 %; b) The accurate classification of sedentary time among adolescents in table 2 range from 96.6 % to 99.5 % (“sitting” and “drawing”, respectively); c) For dancing, table 2 show that 19.9 % was spent in MVPA among adolescents (not 16.9 % as suggested on page 11, line 225). When using 60 seconds epoch length: The lowest accurate classification of sedentary time among children during sitting is while “watching movies” 97.8 % (during “sitting” the figures in table 2 suggest 99.2 %). Also, the numbers seems not to add up to 100 % for the dancing activities among children and adolescents (page 11, lines 225-227). Check and correct these percentages.

AUTHOR’S REPLY

We thank the reviewer for checking these figures and apologize for the mistakes. We have corrected these figures (figures in table 2 are correct). The numbers now add up to 100% for the dancing activity:

Children – 15-second epoch: 24.8% classified as sedentary, 55.1% as LPA and 20.1% as MVPA
Children – 60-second epoch: 38.2% classified as sedentary, 61.8% LPA and 0% as MVPA
Adolescents – 15-second epoch: 19.9% classified as sedentary, 47.1% as LPA and 33.0% as MVPA
Adolescents – 60-second epoch: 25.1% classified as sedentary, 74.9% LPA and 0% as MVPA

Page 13, line 244-245. Since the term “sporadic sedentary time” is introduced, the authors should indicate what this actually means. Referring to the method section and table 3, this should be any sedentary time accumulated in bouts less than 10 minutes. However, I recommend the authors to clarify this anywhere in the method and/or result section and/or table 3 as only “bouts” is used in the table legend (table 2) (this could be interpreted as “short bouts” of sedentary time lasting less than 10 minutes rather than “sporadic sedentary time”).

AUTHOR’S REPLY

We have now defined in the methods section what we mean when referring to sporadic activity, lines 205-207 (page 9):

*Periods less than 10 minutes for sedentary behaviour and LPA and periods less than 5 minutes for MPA, VPA and MVPA were referred to as ‘sporadic’.*

Page 14, table 3. The absolute mean difference for “SB”, “10-29.9 (no tolerance)” do not correspond to the figures provided for 15 seconds vs. 60 seconds epoch length, respectively. Also, a negative sign (minus) appears to be missing for “absolute mean differences” LPA total minutes.

AUTHOR’S REPLY

We apologize for these mistakes. Based on one of the comments of reviewer #1, we critically checked all data and decided to use non-parametric statistics for the analysis of the CHAMPS-data, as most data were not normally distributed. We used medians and 25^th^-75^th^ percentiles to present the data and the Mann-Whitney U test to calculate differences between the different epoch durations, i.e. 5- vs. 15-second epochs, 5- vs. 60-second epochs and 15- vs. 60-second epochs. We carefully checked all numbers and revised our manuscript based on these new analyses:

- Methods, statistical analyses (page 11, lines 237-239): *Using CHAMPS-data, as time estimates according to 5-, 15- and 60-second epoch lengths were not normally distributed, medians (25th - 75th percentiles) were calculated and tested for significance in R using Mann-Whitney U (wilcox.test, R platform 3.4.0; p<0.05).*
- Results: See page 16, lines 289-305.
- Results: Table 3 (pages 17-18) and Figure 1.

Page 17, lines 271-273. In its present form, the reader is not provided with any arguments or information why this epoch length is recommended. The authors should argue why they recommend using a 15-second epoch length.
AUTHOR’S REPLY

We agree with the reviewer that the argumentation for this recommendation was not clear, mainly because we only compared two epoch durations, i.e. 15- and 60-second epochs. We revised this paragraph, now containing the argumentation underlying the 15-second epoch recommendation (page 19-20, 331-340):

*Our findings from the controlled-laboratory study suggests that a 60-second epoch is most appropriate for accurately classifying sedentary behaviour; whereas a 15-second epoch resulted in less dancing time being misclassified as sedentary behaviour than a 5- or 60-second epoch. It should be noted that for sedentary activities misclassifications were relatively small using a 5- or 15-second epoch (i.e. underestimations up to 12% for children and 3% for adolescents), whereas a relatively large percentage of time during dancing was misclassified as sedentary using a 5- or 60-second epoch (i.e. up to 38% for children and 25% for adolescents). It should be noted that we only included one light-to-moderate (dancing) activity. Nevertheless, we recommend using a 15-second epoch when examining both sedentary behaviour and physical activity in children and adolescents.*

Page 17, lines 273-275. The statement “yet it should be kept in mind that applying a 15-second epoch to free-living sedentary behavior may result in 157 minutes more sedentary time per day, compared to a 60-second epoch” needs clarification. The results in table 3 and figure 1a suggest +85.2 minutes per day of sedentary time when using 15 second vs 60 seconds epoch length. If the authors refer to results from another study, a reference should be provided.

AUTHOR’S REPLY

We apologize for the mistake: we were referring to our own data but accidentally copied an incorrect number in the text. In our revised manuscript we deleted this sentence as we no longer used mean differences to compare time estimates of physical activity and sedentary behaviour in the different epoch durations. We kindly refer to our reply in response to a previous comment of the reviewer, including more information on the revised analyses of the CHAMPS-data (i.e. non-parametric statistics).

Pages 18-19, line 289, 294, and 326. Correct from “MPVA” to “MVPA”.

AUTHOR’S REPLY

We thank the reviewer for pointing us to this typo and revised it.

Page 19, line 318. “SB” is used although not previously abbreviated.
AUTHOR’S REPLY

We omitted this abbreviation.

Page 19, line 330. Should “…detection of wear time” be “decision of wear time”?

AUTHOR’S REPLY

We agree with the reviewer that ‘detection’ is not the right wording here and changed this into “… differences in wear time definitions” (page 22, lines 406-409).

Page 19, line 332. Reference for Evenson is number 37 but should (according to the references list) be reference number 5.

AUTHOR’S REPLY

We thank the reviewer for detecting this error and have adjusted it.

Page 21, line 372. The sentence read as follows: “…with a broad classification range, i.e. 101-2295 for a 15-second epoch and 574-1002 for a 60-second epoch”. I guess this would be “26-573 for a 15-second epoch and 101-2295 for a 60-second epoch”?
AUTHOR’S REPLY

The reviewer is correct that 26-573 and 101-2295 are the correct classification ranges. We have adjusted this.

Page 21, conclusion. The statement “…mean differences between 15- and 60-second epochs in free-living accelerometer data were relatively smaller for sedentary behavior than MVPA” is somewhat confusing given that no difference was observed between 15 second vs. 60 second epoch length for MVPA. Should this be “LPA” as the difference was relatively smaller for sedentary behavior than LPA (85.2 min vs. 96.7 min, respectively)? Clarify and revise the conclution.

AUTHOR’S REPLY

We revised the conclusion of our manuscript (page 25, lines 466-474):

*We conclude that the most accurate epoch length seems to be 60 seconds for estimating sedentary time and 15 seconds for physical activity. When examining both physical activity and sedentary behaviour we recommend using a 15-second epoch length, as misclassification of sedentary activities was relatively small using a 5- or 15-second epoch , whereas using a 5- or 60-second epoch resulted in a relatively large amount of physical activity (dancing) misclassified as sedentary behaviour. We further recommend future studies to describe their data reduction procedures in detail. Finally, we recommend studies examining the accuracy of classifying sedentary behaviour, LPA and MVPA to include free-living activities at various intensities.*
